# Effects of lung protective ventilation and conventional ventilation on postoperative atelectasis in neonates under general anesthesia

**DOI:** 10.3389/fped.2026.1730470

**Published:** 2026-02-12

**Authors:** Jingru Wang, Kun Yue, Yingying Sun, Yin Xia

**Affiliations:** Department of Anesthesiology and Perioperative Medicine, Anhui Provincial Children’s Hospital, Hefei, Anhui, China

**Keywords:** anaesthesia, lung-protective ventilation, lung-ultrasound, neonate, pulmonary atelectasis

## Abstract

**Background:**

General anesthesia frequently causes atelectasis, a condition that significantly endangers patient safety during and after surgery, especially in neonates. Evidence suggests that lung protective ventilation (LPV) strategies may reduce the incidence of postoperative atelectasis in patients receiving general anaesthesia; however, the efficacy for neonatal patients remains controversial. This study aims to explore how LPV affects the incidence of atelectasis in neonates.

**Methods:**

This randomized controlled trial involved neonatal patients under general anesthesia with mechanical ventilation for over two hours, randomly assigned to receive LPV (including a Vt of 6 mL/kg, 5 cmH_2_O PEEP, and lung RMs once per hour) or conventional ventilation (including a Vt of 8–10 mL/kg without PEEP or RMs). Each infant underwent two lung ultrasound (LUS) scans at specified time points: 5 min post-intubation and at the conclusion of surgery. Additionally, three arterial blood gas analyses were conducted for each infant at T1 (5 min post-intubation), T2 (one hour after mechanical ventilation), and T3 (two hours after mechanical ventilation). HR and MAP were recorded at four time points: T1, T2, T3, and T4 (at the conclusion of surgery). The primary outcomes were the incidence of significant atelectasis (defined as any area with a consolidation score of ≥2) and LUS scores at the conclusion of surgery.

**Results:**

The study enrolled 100 neonatal patients, divided into 50 in the LPV group and 50 in the control group. At the conclusion of surgery, the LPV group had a significantly lower incidence of significant atelectasis (18% vs. 58%) and lower median LUS scores [7 [6, 9] vs. 12 [8, 18]] than the control group did (all *P* < 0.001). In addition, there were differences in the partial pressure of arterial oxygen (PaO₂) and partial pressure of arterial carbon dioxide (PaCO₂) between the two groups at T2 and T3, although both were within the normal range. No significant differences were observed in HR, MAP or the rate of respiratory events after surgery between the two groups.

**Conclusions:**

Compared with conventional ventilation, LPV results in a significantly lower incidence of significant postoperative atelectasis and lower LUS scores in neonates receiving general anaesthesia.

**Clinical Trial Registration:**

ClinicalTrials.gov, ChiCTR2100051721

## 1 Introduction

Pulmonary atelectasis is defined as the inadequate distension of alveoli and terminal bronchioles, and its typical presentation is marked by the total deaeration of pulmonary units. From a physiological perspective, this condition arises when the collapsing forces generated by positive pleural pressure and surface tension surpass the expanding forces exerted by alveolar pressure and parenchymal tethering (1). Atelectasis is a common complication of the general anaesthesia, particularly in younger patients (2, 3). Younger age (especially in neonates) is associated with a greater risk of intraoperative atelectasis-related hypoxemia, as increased chest wall compliance, reduced pulmonary gas exchange surface, higher metabolic oxygen demand and lower pulmonary oxygen reserve, leading to rapid oxygen desaturation when ventilation is impaired (4, 5). Furthermore, atelectasis occurring during general anaesthesia is associated with reduced lung compliance, compromised oxygenation, elevated pulmonary vascular resistance and postoperative pulmonary complications; these abnormalities can severely impair respiratory function, may persist for more than 24 h postoperatively, and even increase the risk of prolonged hospital stay and mortality in paediatric patients, thereby adversely affecting neonatal clinical outcomes (6–8).

Lung-protective ventilation (LPV) in neonates can mitigate ventilator-induced lung injury through the use of a low tidal volume (Vt), an appropriate positive end-expiratory pressure (PEEP), and lung recruitment manoeuvres (RMs), the objectives of these interventions are to minimize alveolar overdistention, prevent repetitive alveolar collapse and reopening, and alleviate atelectasis (9). In contrast, conventional ventilation practices often involve a Vt of 8–10 mL/kg without the application of PEEP or RMs, which is common in neonates undergoing elective surgery (10–12). The efficacy of LPV in preventing atelectasis is still debated and underresearched, particularly in infants and neonates (13). Furthermore, recommendations for LPV in paediatric patients are still controversial (9). Despite the potential advantages of LPV in adult and paediatric populations, research on its application in neonatal surgeries is lacking. Lung ultrasound (LUS) is a practical, noninvasive, and radiation-free modality that has been increasingly widely applied in routine clinical practice. It can assess pulmonary ventilation and plays a pivotal role in diagnosing paediatric pulmonary diseases, including obstructive and compressive atelectasis of various etiologies, while demonstrating reliable sensitivity and specificity for detecting anaesthesia-induced atelectasis in children (14). Consequently, we conducted a randomized controlled trial to compare LPV with conventional ventilation in neonatal patients who underwent surgical anaesthesia by using LUS.

The aim of this study was to investigate whether compared with conventional ventilation, the LPV technique can reduce the incidence of significant postoperative atelectasis and lower LUS scores in neonates who are receiving general anaesthesia for exploratory laparotomy.

## 2 Materials & methods

### 2.1 Study design, ethical approval, and clinical trial registry

This trial was carried out at Anhui Provincial Children's Hospital in China from April 2022 to December 2024. The trial was registered on October 1, 2021, at http://www.chictr.org.cn/ (trial number: ChiCTR2100051721). Written informed consent was given by all the study participants.

### 2.2 Sample size

The incidence of substantial atelectasis following general anesthesia in neonatal patients has not been previously documented in the academic literature. Therefore, prior to initiating this study, we conducted a preliminary pilot study and calculated the sample size based on lung ultrasound data obtained from that pilot study. The results of this pilot study showed that approximately 55% of cases developed significant atelectasis at the conclusion of surgery. We hypothesized that implementing a protective ventilation strategy could reduce this incidence to 20% (15). According to a power of 0.9 and a two-sided alpha level of 0.05, each group required a sample size of 41 patients. Considering a potential 15% dropout rate, the calculated minimum required sample size was 48 patients per group, and we planned to enroll a total of 100 patients.

### 2.3 Selection criteria

The inclusion criteria for the participants were as follows: signed informed consent; age within one month, with no sex restrictions and a body weight of ≥2.5 kg; scheduled for exploratory laparotomy with an anticipated duration of mechanical ventilation exceeding two hours; and an American Society of Anesthesiologists physical status of I-III. Participants were excluded if any of the following conditions were present: refusal by the parents or legal guardians to participate in the trial; the presence of severe cardiovascular malformations; upper respiratory tract infections; abnormal preoperative chest radiographs; or evident respiratory failure; or if the children were in an extremely poor general condition.

### 2.4 Randomization and blinding

**Figure 1:**
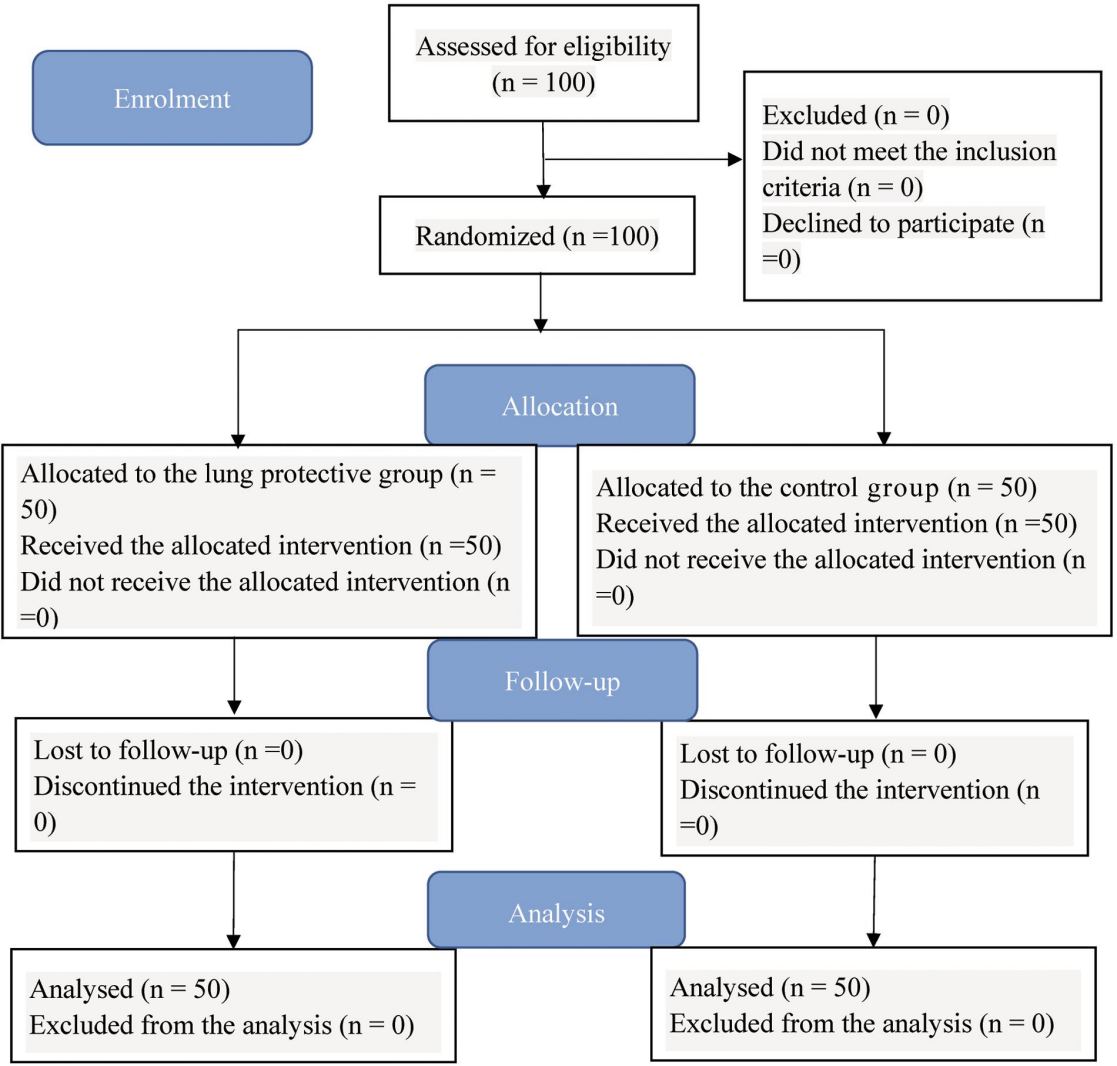
CONSORT study flow diagram.

In this study, participants were randomly assigned to two groups at a 1:1 ratio using a computer-generated sequence, with those assigned odd numbers allocated to the LPV group, and even numbers, to the conventional ventilation group (control group) (Figure 1). The intervention protocols were enclosed in envelopes labelled with these numbers. The task of opening the envelopes and implementing the mechanical ventilation protocols was carried out by a designated anaesthetist. However, the LUS evaluations (5 min post-intubation and at the conclusion of surgery) were conducted by another anaesthetist who was unaware of the grouping of participants and the specific mechanical ventilation protocol implemented for each participant. All involved anaesthetists had undergone strict lung ultrasound evaluation training at the study institute.

### 2.5 Clinical procedure

Specifically, prior to the administration of anaesthesia, anaesthesiologists conducted standard preoperative preparations. A consistent general anaesthesia protocol was applied to all patients, beginning with preoxygenation using 100% oxygen for a duration of 3 min. The induction agents used in this study included midazolam (0.05–0.1 mg/kg), propofol (2–3 mg/kg), sufentanil (0.1–0.2 µg/kg), cisatracurium (0.1 mg/kg), and atropine (0.01 mg/kg). Orotracheal intubation was conducted following the onset of unconsciousness, indicated by the absence of the corneal reflex and jaw relaxation. Mechanical ventilation was administered using a Plus Leon anaesthesia machine. The standard monitoring parameters included heart rate (HR), noninvasive blood pressure (NBP), end-tidal carbon dioxide (P_ET_CO₂), peripheral oxygen saturation (SpO₂), and body temperature. Anaesthesia was maintained using sevoflurane (1%–2%) in combination with an oxygen/air mixture and remifentanil (0.25–0.5 μg/kg/min). Invasive arterial pressure monitoring was used during the surgery. Haemodynamic stability was preserved throughout the surgical procedure, with fluid resuscitation performed according to the surgical conditions and the patients’ vital signs. To reverse the neuromuscular blockade, atropine (0.02 mg/kg) and neostigmine (0.05 mg/kg) were administered, followed by the resumption of spontaneous respiration in the neonates after the surgery was completed (12). Patients were extubated if indicators such as a Vt exceeding 5 mL/kg, intentional movements and the crying reflex were observed (16). Once the patient was moved to the postanaesthesia care unit (PACU), a basic face mask with a reservoir was used to provide them with additional oxygen at a rate of 2–6 L/min, which was slowly decreased to room air as the SpO_2_ levels were >95%. All the patients eventually regained consciousness, had stable circulation, exhibited smooth breathing, showed muscle strength recovery, had a rosy complexion and a strong cough, and reached the standards for leaving the PACU (17). Postoperative follow-up was conducted for 24 h.

**Figure 2:**
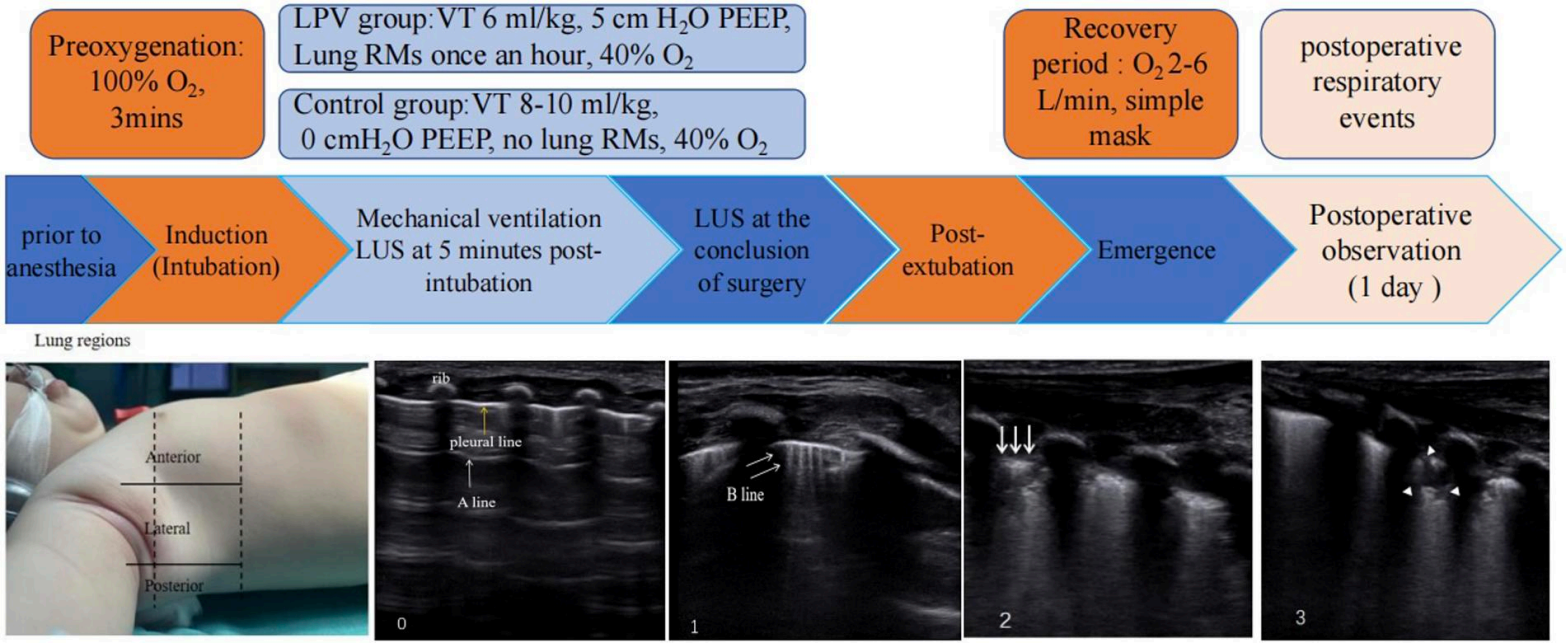
The study protocol and ultrasound lung examination region are shown. From left to right, the scores are as follows: 0, 0–2 B lines; 1, at least three B lines or one or multiple small subpleural consolidations separated by a normal pleural line; 2, multiple coalescent B lines or multiple small subpleural consolidations separated by a thickened or irregular pleural line; and 3, consolidation or small subpleural consolidation of more than 1 cm×2 cm. One yellow arrow, pleural line; one white arrow, A line; two white arrows, B line; three white arrows, subpleural consolidations; white arrowheads, consolidation.

The anaesthetist who was unaware of the grouping of participants performed LUS examinations on each patient at two time points using a device (SONIMAGE HS2—KONICA MINOLTA, Shanghai, China) equipped with a 7–12 MHz linear transducer: 5 min post-intubation and at the conclusion of surgery. In accordance with the protocol by Acosta et al. (14), six regions were delineated in each hemithorax using three lengthwise lines (parasternal, anterior axillary, and posterior axillary) and two crosswise lines (one placed above the diaphragm and the other 1 cm above the nipples). The LUS exam started at the diaphragm and moved to the apex, covering the anterior, lateral, and posterior regions in each hemithorax. Scans of the anterior regions were performed at the midline of the clavicle, scans of the lateral regions at the midaxillary line, and scans of the posterior regions at the 5th and 6th intercostal spaces along the posterior axillary line (18). We assessed and scored each hemithorax using standard two-dimensional views. Each of the 12 regions was identified and assigned an LUS score in accordance with the following scoring criteria (19). Normal aeration (N): 0–2 B lines; small loss of aeration (B1): ≥3 B lines or 1 or multiple small subpleural consolidations separated by a normal pleural line; moderate loss of aeration (B2): multiple coalescent B lines or multiple small subpleural consolidations separated by a thickened or irregular pleural line; complete loss of aeration (C): consolidation or small subpleural consolidation >1 × 2 cm in diameter. Points were assigned according to the worst LUS pattern found in a particular thoracic area: N = 0, B1 = 1, B2 = 2, and C = 3 (Figure 2). Spanning from 0 to 36 for the entire thorax, the LUS aeration score was the cumulative sum of the points from all 12 lung regions; a lower score was considered indicative of greater lung aeration. Moreover, a consolidation score was recorded for each region in accordance with the following scoring criteria (2), with significant atelectasis defined as any area with a consolidation score of ≥2. The degree of juxtapleural consolidation was divided into four grades and scored between 0 and 3: (0) no consolidation; (1) minimal juxtapleural consolidation; (2) small-sized consolidation; and (3) large-sized consolidation.

Mechanical ventilation was configured in pressure-controlled mode with an inspiration-to-expiration ratio of 1:1.5 in both groups. After orotracheal intubation, the fraction of inspired oxygen (FiO_2_) was maintained at 0.4 until extubation, and the ventilatory frequency was set at 20–40/min to maintain 4.7–7.3 kPa of P_ET_CO₂. In the control group, the Vt was set at 8–10 mL/kg without PEEP or RMs (11). Conversely, patients in the LPV group received a Vt of 6 mL/kg and a PEEP of 5 cmH_2_O (20). Simultaneously, in the LPV group, lung RMs was performed according to the recent recommendations (21–23). The lung RMs was performed in pressure control mode, maintaining pressure at a steady 15 cmH_2_O with 5 cmH_2_O increments in PEEP until a peak pressure was achieved 30 cmH_2_O. Patients remained at each incremental PEEP step for 5 s while for 10 s at peak pressure. Then PEEP was reduced in a stepwise manner at the same rate until 5 cmH_2_O and other ventilation parameters were the same as before. During the lung RMs procedure, the vital signs (HR, MAP, and SpO₂) of the neonates were continuously monitored. After the initiation of mechanical ventilation via orotracheal intubation, the LPV group underwent an RMs every hour during mechanical ventilation.

The primary outcomes were the incidence of significant atelectasis (defined as any area with a consolidation score of ≥2) and LUS scores at the conclusion of surgery.

The secondary outcomes included the intraoperative partial pressure of arterial oxygen (PaO_2_), partial pressure of arterial carbon dioxide (PaCO_2_) at T1 (5 min post-intubation), T2 (one hour after mechanical ventilation), and T3 (two hours after mechanical ventilation); HR, mean arterial pressure (MAP), peak airway pressure (Ppeak), dynamic lung compliance (Cdyn) at T1, T2, T3 and T4 (at the conclusion of the surgery); the MAP and HR changes in the LPV group during lung RMs; minimum SpO_2_ during surgery and PACU; extubation time after surgery (defined as the time from the cessation of anaesthetic administration to tracheal tube removal), length of PACU stay (defined as the time from admission to discharge from the PACU), and incidence of postoperative respiratory events (including any that demanded additional care, such as a productive cough with a fever higher than 38 °C, pneumonia, or hypoxia with a pulse oximeter value below 90%) (24).

### 2.6 Statistical methods

Unless stated otherwise, all data are shown as the median or the mean. To evaluate distribution normality, the Shapiro–Wilk test was applied. Mann–Whitney *U*-tests, chi-square tests and repeated-measures ANOVA with a mixed-model procedure were used to assess the outcomes. Statistical significance was indicated by a two-sided *P* value of  < 0.05. SPSS software (ver. 25.0; SPSS, Inc., Chicago, Illinois, USA) and GraphPad Prism (version 9.5.1, Graph-Pad Software, Inc.) were used to conduct the statistical analyses and data presentation.

## 3 Results

### 3.1 Subject characteristics

A total of 100 neonatal patients were finally enrolled in this study, with no patients lost to follow-up; all patients were randomly assigned to the LPV group and the control group, with 50 patients in each group. The entire process of patient recruitment and randomization is summarized in the flow diagram (Figure 1).

There were no differences in patient characteristics or the median duration of mechanical ventilation between the two groups (Table 1). The median LUS scores were similar between the LPV and control groups at 5 min post-intubation [12 [10, 17] vs. 15 [9, 19]; *P* = 0.405], and the incidence of significant atelectasis at this time point was 92% in the LPV group and 88% in the control group, with no statistically significant difference (*P* = 0.505) (Table 2).

**Table 1:** Baseline characteristics and clinical data of neonatal patients under general anaesthesia.

Variables	LPV group (*n* = 50)	Control group (*n* = 50)	*Z/χ^2^*	*P* value
Age/days	2 (1, 4)	2 (1, 4)	−0.124	0.902
Sex(male/female), *n*	35/15	36/14	0.049	0.826
Gestational age at birth/weeks	38 (37, 39)	38 (37,39)	0.000	1.000
Height, cm	49.80 (48.50, 50.90)	50.00 (48.90, 51.00)	−1.080	0.280
Weight, kg	3.08 (2.90, 3.30)	3.01 (2.79, 3.30)	−0.859	0.390
Duration of mechanical ventilation, mins	177.50 (137.00, 210.00)	170.00 (135.00, 205.00)	−0.300	0.764

Values are median (inter-quartile range), *n* (%).

**Table 2:** Comparison of the incidence of significant atelectasis and lung ultrasound scores between the two groups.

Parameters	LPV group (*n* = 50)	Control group (*n* = 50)	*Z/χ^2^*	*P* value
5 min post-intubation				
Total lung ultrasound scores	12 (10, 17)	15 (9, 19)	−0.833	0.405
Anterior regions	1 (1, 2)	2 (1, 2)	−1.363	0.173
Lateral regions	3 (3, 5)	4 (3, 6)	−1.100	0.271
Posterior regions	8 (6, 10)	8 (5, 11)	−0.788	0.431
Incidence of significant atelectasis	46 (92.0%)	44 (88.0%)	0.444	0.505
At the conclusion of surgery				
Total lung ultrasound scores	7 (6, 9)	12 (8, 18)	−4.451	<0.001*
Anterior regions	1 (0, 1)	2 (1, 2)	−3.831	<0.001*
Lateral regions	2 (1, 3)	4 (2, 5)	−4.489	<0.001*
Posterior regions	4 (4, 4)	7 (4, 9)	−4.515	<0.001*
Incidence of significant atelectasis	9 (18.0%)	29 (58.0%)	16.978	<0.001*

Values are median (inter-quartile range), *n* (%).

* *P* value < 0.05.

### 3.2 Primary outcome

At the conclusion of surgery, the LPV group had a significantly lower incidence of significant atelectasis (18% vs. 58%) and lower median LUS scores [7 [6, 9] vs. 12 [8, 18]] compared with the control group, with both differences were statistically significant (both *P* < 0.001) (Table 2).

### 3.3 Secondary outcomes

In each lung region, the median LUS scores significantly differed between the two groups at the conclusion of surgery (anterior regions: 1 [0,1] vs. 2 [1,2], *p* < 0.001; lateral regions: 2 [1,3] vs. 4[2,5], *P* < 0.001; posterior regions: 4 [4,4] vs. 7 [4,9], *p* < 0.001) (Table 2).

**Figure 3:**
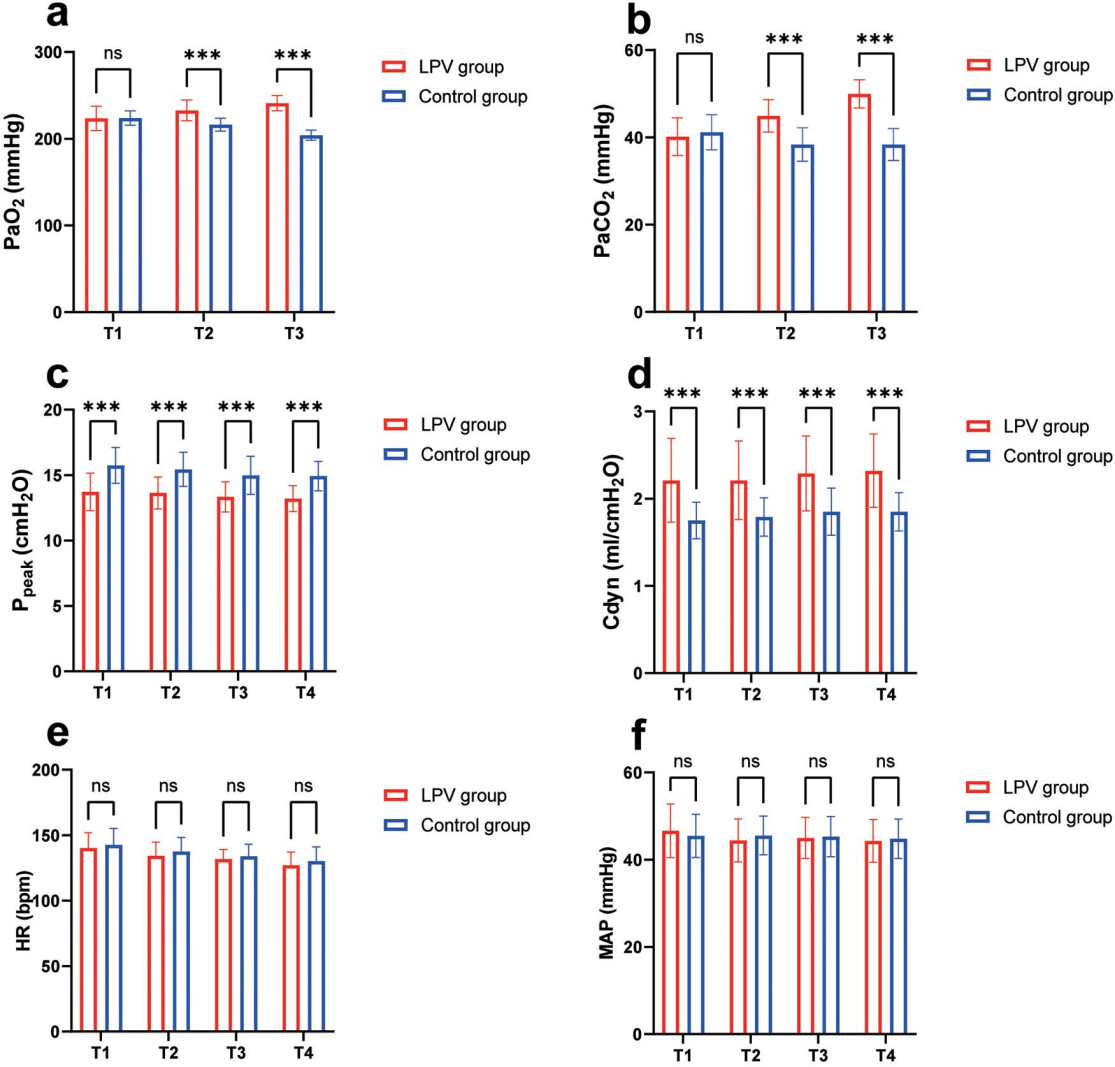
Comparison of intraoperative blood gas analysis, respiratory parameters and vital signs. **(a)** Comparison of PaO_2_ at different time points; **(b)** comparison of PaCO_2_ at different time points; **(c)** comparison of Ppeak at different time points; **(d)** comparison of Cdyn at different time points; **(e)** comparison of HR at different time points; **(f)** Comparison of MAP at different time points; *** indicates statistical significance, *P* < 0.001; ns indicates no significance. T1 (5 min post-intubation), T2 (one hour after mechanical ventilation), T3 (two hours after mechanical ventilation) and T4: at the conclusion of the surgery.

The results of repeated-measures ANOVA revealed that at T1, there were no statistically significant differences in PaO_2_ or PaCO_2_ between the two groups. At T2 and T3, compared with the control group, the LPV group had significantly greater PaO_2_ (232.66 ± 11.93 vs. 216.20 ± 7.45 and 241.02 ± 8.94 vs. 204.07 ± 5.89, respectively) and significantly greater PaCO_2_ (44.94 ± 3.70 vs. 38.36 ± 3.84 and 49.97 ± 3.25 vs. 38.36 ± 3.66, respectively), and all differences were significant (all *P* < 0.001). However, considering the normal range of PaO₂, we consider the result for that parameter to be clinically insignificant (Figures 3a,b).

The results of repeated-measures ANOVA revealed that at T1, T2, T3 and T4, the LPV group had significantly lower Ppeak (13.72 ± 1.43 vs. 15.74 ± 1.37, 13.64 ± 1.22 vs. 15.44 ± 1.30, 13.34 ± 1.15 vs. 14.98 ± 1.45, 13.20 ± 0.99 vs. 14.92 ± 1.12) and significantly greater Cdyn (2.21 ± 0.48 vs. 1.75 ± 0.21, 2.21 ± 0.45 vs. 1.79 ± 0.22, 2.29 ± 0.43 vs. 1.85 ± 0.27, 2.32 ± 0.42 vs. 1.85 ± 0.22) than the control group did, with all the differences being statistically significant (*P* < 0.001) (Figures 3c,d). In addition, the results of repeated-measures ANOVA revealed that there was no significant difference in HR or MAP between the two groups at T1, T2, T3 and T4 (Figures 3e,f).

**Figure 4:**
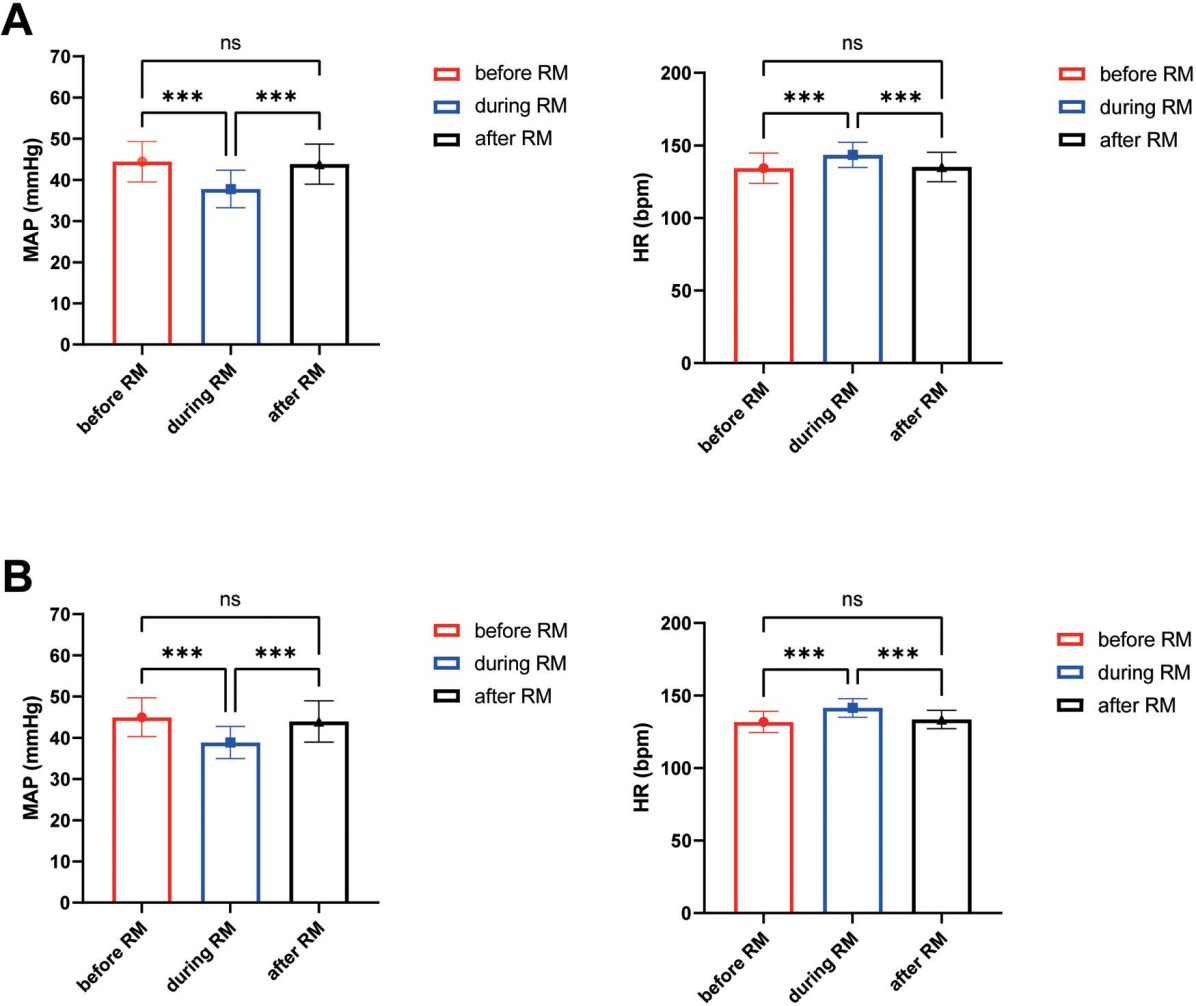
The circulatory changes in the LPV group during lung RMs. **(A)** Circulatory changes during the first lung RMs; **(B)** Circulatory changes during the second lung RMs; *** indicates statistical significance, *P* < 0.001; ns indicates no significance.

The results of repeated-measures ANOVA revealed that in the LPV group, MAP decreased significantly during the first lung RMs compared with the before RMs baseline (37.78 ± 4.57 vs. 44.42 ± 4.92; *p* < 0.001), and returned to baseline levels after the RMs was completed (43.82 ± 4.88 vs. 44.42 ± 4.92; *p* > 0.05). HR increased significantly during this same RMs episode compared with the before RMs baseline (143.48 ± 8.63 vs. 134.32 ± 10.43; *p* < 0.001), and returned to baseline levels after the RMs was completed (135.12 ± 10.19 vs. 134.32 ± 10.43; *p* > 0.05). In addition, MAP decreased significantly during the second lung RMs compared with the before RMs baseline (38.84 ± 3.90 vs. 44.98 ± 4.71; *p* < 0.001), and returned to baseline levels after the RMs was completed (43.96 ± 5.01 vs. 44.98 ± 4.71; *p* > 0.05). HR increased significantly during this same RMs episode compared with the before RMs baseline (141.48 ± 6.42 vs. 131.76 ± 7.30; *p* < 0.001), and returned to baseline levels after the RMs was completed (133.42 ± 6.37 vs. 131.76 ± 7.30; *p* > 0.05) (Figures 4A,B).

The extubation time [35 [32,38] vs. 43.5 [36,48]; *p* < 0.001] and PACU stay [38.5 [35,45] vs. 45 [38,50]; *p* < 0.001] were significantly lower in the LPV group than in the control group. There was no significant difference in the incidence of postoperative respiratory events between the LPV group and the control group (12% vs. 16%; *p* = 0.564). There was also no significant difference in minimum SpO_2_ during surgery or minimum SpO_2_ in the PACU (Table 3).

**Table 3:** Comparison of intraoperative and postoperative oxygenation, recovery time, and postoperative respiratory events rates between the two groups.

Parameters	LPV group (*n* = 50)	Control group (*n* = 50)	*t*/*χ*^2^	*P* value
Extubation time (min)	35 (32, 38)	43.50 (36, 48)	−4.587	<0.001*
Minimum SpO_2_ during surgery (%)	98 (97, 99)	98 (96, 99)	−1.332	0.183
Minimum SpO_2_ in PACU (%)	98 (97, 99)	97 (96, 98)	−1.496	0.135
PACU stay (min)	38.50 (35, 45)	45 (38, 50)	−3.605	<0.001*
Postoperative respiratory events (%)	6 (12%)	8 (16%)	0.332	0.564

Values are median (inter-quartile range), *n* (%).

* *P* value < 0.05.

## 4 Discussion

The results of this randomized and controlled study revealed that compared with conventional ventilation, LPV with a low Vt (6 mL/kg), 5 cmH_2_O PEEP, and lung RMs significantly reduced the incidence of significant postoperative atelectasis and lower LUS scores in neonates after exploratory laparotomy, and no significant differences in vital signs were observed between the groups during mechanical ventilation. However, the rate of respiratory events after surgery was similar among neonates regardless of the ventilatory strategy used.

There are limited clinical data regarding LPV strategies in neonates undergoing elective surgery. One review recommended avoiding the use of high Vt, reversing atelectasis, stabilizing lung units during both inspiration and expiration, and employing lower oxygen concentrations as a rational approach (25). Previous research has indicated that using LPV strategies, such as a low Vt (26), PEEP (26, 27), and lung RMs (22), can effectively prevent atelectasis in paediatric patients. Given the young age of our patient cohort, higher levels of PEEP could have led to haemodynamic instability; therefore, we implemented a lower PEEP threshold of 5 cm H_2_O within the LPV framework (28). Moreover, we incorporated lung RMs as part of the LPV strategy (29). Although the RMs has several benefits, it has also been associated with side effects such as hypotension, SPO_2_ decrease, barotrauma, and lung injury caused by the ventilator; however, the reported incidences are very low (23). Although RMs may induce circulatory changes, we dynamically monitored invasive arterial blood pressure. For safety reasons, if the blood pressure decreased by more than 20%, we stopped further PEEP elevation or reduced the PEEP and repeated these adjustments (22). Our data revealed that both HR and MAP underwent changes during lung RMs; however, they gradually returned to pre-RMs baseline levels following completion of the RMs. In the application of RM in newborns, we should pay close attention to the change of circulation, so that this technology can be safely applied to anesthesia in neonatal surgery.

Anaesthesia often leads to atelectasis (1, 30). The study by Gao et al. revealed that atelectasis appeared in infants under three months of age after anaesthesia induction (29). We observed that at 5 min post-intubation, approximately 85% of patients (91% in the LPV group and 88% in the control group) developed significant anaesthesia-induced atelectasis. We present the initial objective evidence indicating that anaesthesia-induced neonatal atelectasis occurs as early as 5 min after the initiation of mechanical ventilation. Prior investigations have shown that significant atelectasis occurs in approximately 60%–80% of patients after anaesthesia induction, a figure lower than the baseline LUS observed in this study (2, 29, 31). We believe that this difference is mainly due to the inclusion of a relatively younger population in our study. According to a prior study, there appears to be a negative correlation between age and atelectasis formation, indicating that younger age is associated with a greater incidence of atelectasis (31). The unique characteristics of neonatal lung physiology, such as a highly compliant chest wall and a closing volume higher than the functional reserve capacity (FRC), may make neonates more prone to atelectasis (2). Therefore, this confirms what we suspected. Owing to the high oxygen consumption of neonates and their poor ability to tolerate hypoxia, the oxygen reservoir needs to be increased during tracheal intubation. In our study, we used positive-pressure ventilation to administer 100% oxygen for preoxygenation, which may be the reason that atelectasis occurred in the first few minutes. However, to our knowledge, the incidence of significant atelectasis is similar whether preoxygenation is performed with 60% or 100% oxygen during induction (32). A previous study suggested that the reduction in continuous positive airway pressure and the decrease in FRC during tracheal intubation could be a key factor in the formation of notable atelectasis after anaesthesia induction (33).

Various studies have indicated that LPV reduces postoperative pulmonary complications, improves lung gas exchange, and reduces hospital stays in adults and children (11, 34–36). The results of the present study were consistent with those of prior studies involving children (15, 33, 37). The study (12) involving children indicated that LPV is more effective at preventing atelectasis during and after surgery; our results align with those of that study but are from a younger cohort. LPV encompasses more than merely low Vt ventilation; it is important to acknowledge that low-Vt ventilation alone may result in the cyclic opening and closing of ventilated alveoli adjacent to collapsed ones, thereby increasing shear stress-related damage to the distal airways and alveoli (38). Presently, LPV is understood to be based on two additional fundamental principles (11). First, it involves maintaining lung openness through lung RMs to prevent a decrease in lung volume. Second, it requires the prevention of cyclic alveolar collapse by employing personalized and appropriately adjusted PEEP ventilation. The advantages of RMs with PEEP underscore the need for early, active management during mechanical ventilation in neonates (2). A significant relationship exists between atelectasis, a decrease in SPO_2_, and a decrease in PaO_2_ and poor clinical outcomes (39). Our research revealed that atelectasis is more likely to occur in the lateral and posterior regions. Moreover, Jang reported that atelectasis occurs unevenly in various lung regions during general anaesthesia, with a greater frequency in the lateral and posterior regions (33). These findings align with those of our study. This phenomenon may be attributed to inadequate ventilation in the lateral and posterior regions of the lungs during anaesthesia and in the supine position. Large-scale studies across multiple centres are needed to explore the clinical importance of LPV in neonates under general anaesthesia.

This study is subject to several limitations. First, the sample was restricted to healthy neonates with normal respiratory and circulatory physiology, and those with severe underlying conditions or compromised general health were excluded. Neonates with such conditions may require more precise monitoring and intervention to mitigate the effects of anaesthesia on their respiratory and circulatory systems. Second, LUS is a semiquantitative tool, and its results are dependent on the operator's expertise. To address this, an experienced anaesthesiologist conducted all the examinations, while a separate researcher who was uninvolved in the ultrasound scanning performed the scoring of all the ultrasound images. This study focused on neonates, a population for whom LUS examinations are relatively straightforward. Third, we did not determine whether complete resolution of atelectasis is required for improving postoperative outcomes; this question should be addressed in future research.

## 5 Conclusions

In conclusion, our study suggests that for anaesthesia for neonatal surgery, the LPV technique, combining low Vt, PEEP and lung RMs, is a feasible and safe approach. The results suggest that in contrast to conventional ventilation, LPV induces a reduction in the incidence of significant postoperative atelectasis and lower LUS scores after exploratory laparotomy in neonates who are receiving general anaesthesia. This study provides a basis for the application of LPV techniques in anaesthesia administration for neonatal surgery.

## Data Availability

The original contributions presented in the study are included in the article/Supplementary Material, further inquiries can be directed to the corresponding authors.
